# Exploring linguistic features and user engagement in Chinese online mental health counseling

**DOI:** 10.1016/j.heliyon.2024.e38042

**Published:** 2024-09-20

**Authors:** Liyuan Zhang, Dexi Liu, Jing Li, Changxuan Wan, Xiping Liu

**Affiliations:** a School of Computer and Artificial Intelligence, Jiangxi University of Finance and Economics, Nanchang, 330013, JiangXi, China; b School of Mathematics and Computer, YuZhang Normal College, Nanchang, 330013, Jiangxi, China; c Jiangxi Key Laboratory of Data and Knowledge Engineering, Jiangxi University of Finance and Economics, Nanchang, 330013, Jiangxi, China; d School of Electronic Management Science, Fujian Jiangxia University, Fuzhou, 350108, Fujian, China

**Keywords:** Online mental health counseling, The topics of questions and answers, Vocabulary categories, Linguistic style matching, Language modeling

## Abstract

With the popularity of online mental health platforms, more individuals are seeking help and receiving social support by openly discussing their problems. Therefore, it's crucial to gain a deeper understanding of which problem disclosures and social support on these platforms can attract more user attention and engagement. Previous research has primarily focused on social media forums. Our work concentrates on the professional mental health platform, intending to understand the linguistic features present in posts that promote user engagement and interaction. We employ text mining and deep learning techniques to analyze posts consisting of 22,250 questions from help-seekers and 78,328 answers providing social support extracted from the Chinese online mental health counseling platform. Initially, we analyze the high-frequency words and topics of the questions and answers to gain insights into the primary focal points and the range of topics covered in these posts. The results indicate that work-related issues are the most concerning and troublesome for help-seekers, and the topics that users follow are approximately 8 types, including growth, family, in-love, marriage, emotions, human-relations, behavioral-therapy and career. Subsequently, we analyze the language usage in question-and-answer posts with different engagement from three aspects: vocabulary categories, linguistic style matching, and language modeling, aiming to identify which linguistic features can attract more user attention and engagement. The results reveal that high-engagement answer posts exhibit a higher degree of linguistic style matching with the corresponding questions, and the use of vocabulary categories also influences the attention and engagement of the posts. By exploring the linguistic features and patterns displayed in posts with different levels of engagement on the professional online mental health platform, this study offers deep insights into user behavior and the factors that impact counseling effectiveness on the platform and provides valuable knowledge for understanding effective user interactions and engagement.

## Highlights

• We construct a Chinese mental health dataset consisting of questions and answers provided by help-seekers and psychological supporters, which comprises single-turn Q&A dialogues with the form of long-text.
• We discover that on the professional Chinese online mental health counseling platform, users' questions are mainly in eight categories: human-relations, family, marriage, career, emotions, in-love, behavioral-therapy, and growth, with work issues being the top concern.
• We find that high-engagement answers on the Chinese mental health platform show better linguistic style matching to the questions. The use of specific vocabulary categories also influences the attention and engagement of Q&A posts.

## 1 Introduction

According to the latest data from the World Health Organization, there are approximately 450 million individuals worldwide with mental health disorders [1]. In 2021, a mental health survey in China showed that approximately 173 million people were experiencing mental illnesses, such as anxiety, depression, and obsessive-compulsive disorder. However, over 150 million have never sought professional treatment [2]. Mental health issues are becoming increasingly severe and have a significant impact on human health and society. With the widespread use and convenience of social media, more individuals are turning to online platforms to express their emotions or seek help when faced with emotional distress. The convenience and anonymity of social media have resulted in a plethora of question-and-answer posts that not only contain the emotional expressions of help-seekers but also showcase the professional knowledge offered by social supporters [3]. Existing research has focused on these posts, including studies on help-seekers self-disclosure willingness and its influencing factors [4], the relationship between user language signals, emotional polarity, post length, and social support [5], [6], [7], as well as the behavioral characteristics and dynamic changes of user roles [8]. Researchers have also investigated factors influencing the types of social support users receive [9] and analyzed the impact of the content of social support on its effectiveness [10], [11].

While existing research has provided some analysis of these posts, the current literature has certain limitations. Firstly, existing literature primarily focuses on user behavior and content from social networks and lacks sufficient attention to professional online mental health counseling platforms. Social networks encompass a wide range of users and types of questions, not all of which are related to mental health, and the answers provided by ordinary users often lack expertise. In contrast, professional mental health counseling platforms primarily focus on help-seekers emotional distress, and the social supporters are mostly qualified psychologists or counselors who can provide more professional and helpful responses. Previous research has indicated that help-seekers prefer to seek useful information from highly reputable mental health professionals compared to ordinary supporters [12]. Therefore, further research is needed to explore the data from professional online mental health counseling platforms to gain a deeper understanding of user interaction behaviors and outcomes on these platforms. Additionally, there is a lack of sufficient research on the features and differences between posts that receive different levels of attention and engagement on professional online mental health counseling platforms. While social supporters on professional mental health platforms are mostly qualified psychologists or counselors, our observations indicate that there are still many answer posts that are simplistic, repetitive, and stereotypical, lacking specificity and helpfulness, thus failing to effectively foster user identification and engagement. Therefore, an important issue to address is what features or factors contribute to posts receiving higher user attention and engagement on these platforms. Such analysis can help us understand effective interactions among users and provide positive social support to help-seekers [11].

This study selects the “Mental Health Q&A” sub-forum of the well-known Chinese online mental health platform “壹心理” (Yi Xinli)^1^ as the data source. “Yi Xinli” is an online platform dedicated to mental health services, with nearly 20 million users, 7,000 mental health experts, and column authors, helping over 500 million people overcome psychological difficulties. The platform allows users to communicate, share, and interact on various psychological issues, and the social supporters are qualified psychological counselors. “Yi Xinli” focuses specifically on mental health, which means that its users are more inclined to seek help and support in the field of psychology. Therefore, “Yi Xinli” can more accurately reflect the psychological concerns and situations of help-seekers compared to other social platforms. Additionally, the platform's provided psychological counseling services are more professional and targeted [13]. In summary, the data from this platform can comprehensively and accurately represent the real situation in the field of psychological issues, making it worthy of in-depth research.

We collect 22,250 question posts and the corresponding 78,328 answer posts from the “Mental Health Q&A” sub-forum of the “Yi Xinli” platform. The question posts are from help-seekers, and the answer posts are from social supporters who are experts or counselors with psychological qualifications. We employ text mining techniques and LDA topic modeling to analyze the high-frequency words and topics in these posts. The results reveal that work-related issues are the primary concerns and challenges for help-seekers. Additionally, the question topics of help-seekers primarily encompass eight categories: human-relations, family, marriage, career, emotions, in-love, behavioral-therapy, and growth. Furthermore, we conduct analyses in three aspects: vocabulary categories, linguistic style matching, and language modeling, to uncover the linguistic usage features and differences in question-and-answer posts with different levels of engagement. The findings demonstrate that high-engagement answer posts exhibit a higher degree of linguistic style matching with the corresponding questions, and the use of vocabulary categories also influences the attention and engagement levels of the posts.

This paper's primary contributions are as follows: (1) We construct a Chinese Q&A dataset of online mental health counseling, which includes question posts seeking psychological support and answer posts providing psychological support. The questions and answers are presented in the form of long texts, covering a wide range of question topics. Moreover, the answer content is both professional and comprehensive. The dataset provides diversified data samples for research and applications in the field of mental health, particularly supporting studies related to long-text automatic Q&A in the Chinese context. (2) We discover that on the professional Chinese online mental health counseling platform, users focus on eight categories of question topics, with work-related issues being the most distressing and concerning. These findings intuitively reveal the reasons and focal points of psychological problems faced by current Chinese mental health users. (3) We find that high-engagement answer posts exhibit a higher degree of linguistic style matching with the corresponding questions. Additionally, the utilization of specific vocabulary categories also influences the level of attention and engagement received by the question-and-answer posts.

This study examines the linguistic features and patterns observed in posts with different levels of engagement on the Chinese professional mental health platform, providing valuable insights into user behavior and the factors influencing counseling effectiveness. The findings highlight the critical role of linguistic style and vocabulary usage in promoting user engagement, thereby deepening our understanding of effective user interaction on the professional platform. These findings have practical implications for improving the service quality of online mental health counseling platforms, promoting active user engagement, and delivering effective support to users.

## 2 Related work

**Support in Online Mental Health Platforms.** With the popularity and anonymity of the Internet, an increasing number of users choose to disclose their mental health issues on social platforms and seek psychological support [12], [14]. Many social platforms provide dedicated Q&A sections for users to seek psychological counseling. For example, Reddit has a mental health section, 7Cups offers discussion forums under specific conditions, and Talklife has interactive sections based on emotional issues [15]. The Chinese social platform “Weibo” also has a specialized section for psychological questions, where users can share their psychological problems and provide help [16]. Additionally, professional psychological service platforms are targeting specific mental disorders, such as platforms for anxiety and depression [17], [18], as well as platforms focusing on mental health, suicidal ideation, and early intervention for young people [19]. Chinese online platforms for psychological counseling, such as “Yi Xinli” and China Mental Health Net,^2^ also provide professional psychological support to users. Extensive research has explored these platforms. Wetterlin et al. [20] examined the experiences of young people using traditional and online mental health resources, as well as their expectations of mental health platforms. Blair et al. [21] proposed suggestions for supporting constructive mental health discussions on Instagram to effectively assist users facing mental health issues. Sharma et al. [22] conducted a large-scale analysis of engagement patterns of mental health users on TalkLife and Reddit, revealing the roles, limitations, and designs of these platforms. Furthermore, Gandhi et al. [23] investigated mental health discussions in live-streaming gaming communities to understand perceptions of mental health discussions in that context. Participating users in online mental health services can obtain information and emotional support from supporters, thereby improving their health status and strengthening self-health management [24], [25].

**The Influence of Language Use.** The language use of users can impact the results of counseling sessions. Numerous scholars have analyzed the language use of users to identify significant information in social networks [26], [27], [28]. In terms of the language use of help-seekers, Coppersmith et al. [6] classified disclosure data of four mental disorders on Twitter, and found that patients with depression significantly increased their use of negative emotional words and first-person pronouns, while patients with other disorders significantly increased their use of profanity and angry words. Liu et al. [26] analyzed self-disclosures with suicidal thoughts and behaviors from the Chinese social network Weibo and discovered that the frequency of death-oriented words used by users significantly decreased, while the frequency of future-oriented words significantly increased after counseling. Jiang et al. [29] analyzed the impact of language use of help-seekers on communication and social support in the Chinese health community “Tianmijiayuan” and found that different vocabularies, sentence readability, conciseness, and length can all affect the social support received, and lexical richness in health-related vocabulary negatively correlates with receiving informational social support. In terms of the language use of social supporters, Saha et al. [28] examined the language use of social supporters on TalkLife and discovered that supporters' complex language use, including adaptability, diversity, and emotionality, were crucial factors in driving positive psychological changes in help-seekers. In contrast, other simple factors, such as the number and immediacy of support, had no significant impact on mental health improvement. Chikersal et al. [30] studied the various support modes and strategies utilized by social supporters in different contexts in SilverCloud and found that the supportive feedback directly influenced the beneficial outcomes for help-seekers.

**Topic Analysis.** Analyzing the topics of users' posts can provide a summary and understanding of users' needs, behaviors, states, and the background of problems [31], [32]. Scholars have analyzed the topics of mental health patients' posts to uncover pertinent information. For instance, Sik et al. [31] modeled the posts in depression forums and observed that individuals expressed their depression in varying ways under different topics. Park et al. [33] investigated the posts on Reddit and found that the depression group primarily focused on self-expression related to depression, whereas the anxiety and post-traumatic stress disorder groups placed more emphasis on treatment-related issues. Joseph et al. [34] employed semantic communities and topic modeling techniques to analyze suicide notes and mental health posts from Reddit and gain insights into the emotions and topics expressed within these posts. The majority of previous research has centered on analyzing the subjects of question posts made by help-seekers, with less attention given to the topics of answer posts from social supporters. A limited number of studies have categorized social support methods by examining the topics of answer posts. For instance, De & De [4] classified social support types into four categories: emotional support, informational support, instrumental support, and normative support, and compared the distinctions between each type. Based on this, Shavazi et al. [35] expanded the social support categories to five: informational support, emotional support, network support, respect support, and tangible support.

**User Engagement.** With the rise of social media platforms, an increasing number of studies have focused on issues related to user engagement. Scholars argue that user engagement is a mutually dependent process, where users actively contribute to social platforms by sharing personal information, experiences, and knowledge, highlighting the interactive, bidirectional, and behavioral nature of engagement [36]. Shah et al. [37] found that rational, emotional, and transactional content positively impacts user engagement. Feng et al. [38] discovered that community factors, such as social identification and perceived effectiveness, play a significant role in influencing user engagement. Furthermore, researchers have studied factors influencing the engagement of social supporters on Instagram, revealing that informational support has a positive impact on user engagement, while the influence of vocabulary on engagement varies depending on the social supporter's identity [39]. Li et al. [40] found that users benefit from emotional and informational social support, leading to an increased willingness and behavior of sharing personal information on social platforms. Chikersal et al. [30] analyzed the behavioral characteristics of supporters based on user outcomes and found that specific, positive, and supportive feedback encourages active user engagement.

Overall, existing research has shed light on various aspects related to support platforms, language use, topic analysis, and user engagement in online mental health counseling. However, existing research mainly focuses on data from social networks and lacks sufficient attention to professional online mental health counseling platforms. Moreover, there is a lack of sufficient research on which factors can influence users to gain more attention and engagement on these professional platforms. This study focuses on the professional Chinese online mental health counseling platform, aiming to explore the linguistic features and differences of obtaining different engagement Q&A posts on the platform.

## 3 Methods and results

### 3.1 Dataset

The dataset used in this study is sourced from the “Mental Health Q&A” sub-forum of the “壹心理 (Yi Xin Li)” platform, covering the period from November 2011 to October 2022. The dataset is presented in single-turn Q&A format, where each question has multiple diverse answers, and each answer includes the number of “likes” generated by the “like” tag, on which users click to express their recognition of the answer. The questions are posed by users seeking psychological assistance, while the answers are provided by qualified psychological counselors, offering helpful responses to the users' questions. Moreover, both the questions and answers are presented in the form of long texts. Non-textual content such as empty values, line breaks, spaces, emoticons, and advertising links, has been removed. To ensure data quality, only questions with at least one reply were selected. Due to the majority of answer posts being lengthy texts, we excluded shorter answers and only selected those with at least 100 characters. Ultimately, we obtain a total of 22,250 question posts and 78,328 answer posts. The basic information of the dataset is shown in Table 1.

**Table 1:** The basic information of “Yi Xinli” question-and-answer dataset.

Statistical content	Number
number of questions	22250
number of answers	78328
the average/median length of question title (characters)	21.5/ 23
the average/median length of question content (characters)	238.5/ 216
the average/median length of answer content (characters)	570.4/ 472
the average/maximum “likes” received by answers	4/ 53
the average/maximum replies received by questions	3.6/ 119

### 3.2 Focal points and topics of questions and answers

To gain insight into the focal points and range of topics of questions and answers, we analyze them from two perspectives: high-frequency words and topics.

#### 3.2.1 High-frequency words

Words are the carriers of content, and high-frequency words can reflect the focus content of questions and answers. Table 2 shows the high-frequency words of question posts by using jieba word segmentation^3^ to segment, remove stop words and then count the word frequency (number of occurrences of the words). The result indicates a substantial number of emotional words in question posts, such as “like,” “fear,” “anxiety,” “painful,” “uncomfortable,” and “depressed”, with the majority relating to “work,” “friends,” “parents,” “children,” “relationships,” “studying,” “in love,” and “family”, among which “work” is particularly prominent.

**Table 2:** The word frequency counting of question posts.

Word	Frequency	Word	Frequency	Word	Frequency
感觉 (sensation)	12914	希望 (hope)	3066	同学 (classmate)	2367
喜欢 (like)	9238	焦虑 (anxiety)	3053	学校 (school)	2315
**工作 (work)**	8379	发现 (find)	3021	性格 (character)	2315
情绪 (emotion)	7041	老师 (teacher)	2891	内心 (heart)	2272
**朋友 (friends)**	6765	影响 (influence)	2723	父亲 (dad)	2270
**父母 (parents)**	5994	好像 (seem)	2695	压力 (pressure)	2252
母亲 (mum)	5393	时间 (time)	2679	想要 (want)	2213
**孩子 (children)**	5126	说话 (talk)	2666	想法 (idea)	2172
不好 (bad)	5007	家里 (home)	2583	难受 (uncomfortable)	2155
生活 (life)	4845	成长 (growth)	2575	情况 (situation)	2151
害怕 (fear)	4443	女生 (girl)	2534	男朋友 (boyfriend)	2097
**关系 (relationship)**	4241	分手 (break up)	2522	抑郁 (depressed)	2092
**学习 (learning)**	3938	结婚 (marriage)	2512	男生 (boy)	2090
心理 (psychology)	3731	治疗 (treatment)	2436	大学 (college)	2089
**恋爱 (in love)**	3604	痛苦 (painful)	2410	相处 (get along with)	2076
**家庭 (family)**	3339	感情 (feeling)	2389	人际关系 (human relations)	2070

Similarly, we count the word frequency on answer posts. The results show that many of the common words in answer posts are similar to those in question posts, but also include words unique to answer posts, such as “hello,” “advice,” “description,” “acceptance,” “needs,” “cognition,” “perhaps,” “try,” “think,” “method,” “hug,” and other words used to express emotional support, guidance, advice, or empathy.

#### 3.2.2 Topics

LDA (Latent Dirichlet Allocation) is one of the most mature and representative models for topic analysis. It is a probabilistic generative model that effectively captures the latent thematic structure within a corpus [41]. LDA has been widely applied in various domains, such as social media text analysis, and has successfully extracted topics from textual data [42], [43]. In this paper, we utilize the LDA model to analyze the topics of questions and answers. Initially, we use the questions and answers text data as the input for the LDA topic model and segment the text data using jieba word segmentation. After segmentation, we vectorize the texts using the doc2bow bag-of-words model in gensim, calculate word frequency using the TfidfModel in gensim, and obtain the final vectorized representation of questions and answers. Subsequently, we employ the LdaModel package in gensim to model the vectorized texts of questions and answers. Finally, we use the pyLDAvis package to visualize the results of the topic analysis.

After making repeated adjustments to the visualization results and considering the consistency and perplexity of the topics (higher consistency and lower perplexity indicate better results) for the question posts, we selected the number of topics as 8 when the distribution of each topic is relatively scattered and averaged in the visualization results, indicating clear distinctions between each topic and relatively balanced numbers of documents for each topic. After 40 iterations and multiple clustering, we obtain the LDA topic model visualization results for the question posts, as shown in Appendix A.

By analyzing the distribution of top-30 most relevant keywords in the 8 topics, such as the keyword sequence “孩子 (children)-老公 (husband)-东西 (stuff)-解决 (solve)-离婚 (divorce)-人生 (life)-家庭 (family)...” in topic 3 and the keyword sequence “分手 (breakup)-性格 (personality)-恋爱 (in love)-男朋友 (boyfriend)-交往 (dating)-见面 (meeting)-合适 (suitable)-痛苦 (pain)-怀疑 (doubt)-相处 (get along)...” in topic 6, the types of questions expressed in each topic are relatively clear. Therefore, based on the distribution of keywords, we classify the topics of the question posts into 8 categories: “human-relations,” “family,” “marriage,” “career,” “emotions,” “in-love,” “behavioral-therapy,” and “growth”, as shown in Table 3.

**Table 3:** Distribution of top-30 most relevant keywords (partial) in the topics of question posts.

Topic	Keyword sequence
human-relations	异性 (the opposite sex)-朋友 (friend)-室友 (roommate)-人际交往 (human relations)-逃避 (escape)-大学 (college)-纠结 (conflict)...
family	爸爸 (dad)-妈妈 (mum)-出轨 (be derailed)-吵架 (quarrel)-怀疑 (doubt)-家里 (home)-父母 (parents)-失眠 (insomnia)...
marriage	孩子 (children)-老公 (husband)-东西 (stuff)-解决 (solve)-离婚 (divorce)-人生 (life)-家庭 (family)...
career	公司 (company)-厌恶 (detest)-加班 (work overtime)-变得 (become)-迷茫 (confused)-崩溃 (breakdown)-逃避 (escape)...
emotions	痛苦 (pain)-情绪 (emotion)-精神 (spirit)-改变 (change)-内心 (heart)-接受 (accept)-失败 (fail)-放弃 (give up)-发脾气 (get angry)...
in-love	分手 (breakup)-性格 (personality)-恋爱 (in love)-男朋友 (boyfriend)-交往 (dating)-见面 (meeting)-合适 (suitable)-痛苦 (pain)-怀疑 (doubt)-相处 (get along)...
behavioral-therapy	自残 (self-mutilation)-自杀 (suicide)-矛盾 (contradiction)-抑郁症 (depression)-紧张 (nervous)-情绪 (emotion)-低落 (downcast)-药物 (drug)-治疗 (treatment)...
growth	高中 (high school)-小时候 (childhood)-高考 (college entrance examination)-成绩 (achievement)-压力 (pressure)-负面 (negative)-考试 (exam)-焦虑 (anxiety)...

By examining the keywords corresponding to each topic, we can discern the primary content expressed in question posts and deduce the underlying causes or contexts of mental health issues, such as the keywords “work overtime,” “confused,” and “breakdown” in career topic, and the keywords “high school,” “college entrance examination,” and “achievement” in growth topic.

Similarly, we apply LDA model to analyze answer posts, following the same process as question post topic analysis. After 40 iterations and multiple clustering, we set the number of topics to 4 for answer posts, and the results are shown in Appendix B. Analysis of the distribution of top-30 most relevant keywords reveals that the content boundaries of each topic in answer posts are not clear, and it is difficult to roughly classify the topic categories through keywords like question posts. Therefore, this study does not summarize the topic categories for answer posts. Table 4 illustrates the distribution of keywords for each topic in answer posts.

**Table 4:** Distribution of top-30 most relevant keywords (partial) in the topic of answer posts.

Topic	Keyword sequence
topic1	孩子 (children)-妈妈 (mum)-爸爸 (dad)-婚姻 (marriage)-男友 (boyfriend)-担心 (worried)-朋友 (friend)-痛苦 (pain)-关心 (care)-心理咨询 (psychological counseling)-计划 (plan)...
topic2	学校 (school)-面对 (face)-稳定 (stabilize)-自卑 (inferiority)-自我 (self-centered)-心态 (mentality)-能量 (energy)-逃避 (escape)-认可 (approve)-迷茫 (confused)-调节 (adjust)...
topic3	职业 (career)-支持 (support)-交往 (dating)-寻找 (seek)-理想 (ideal)-渴望 (desire)-经验 (experience)-表达 (express)-试着 (try)-咨询 (counseling)...
topic4	爱情 (love)-分手 (break up)-感受 (feeling)-害怕 (fear)-抱抱 (hug)-温暖 (warm)-勇敢 (brave)-小时候 (childhood)...

Based on the analysis of keyword sequence in the topics of both answer posts and question posts, some similarities can be found, such as “children,” “escape,” “confused,” and “pain”. However, there are also obvious differences. For instance, emotional symptoms (such as “anxiety,” “self-mutilation,” and “suicide”) are significantly reduced, while some emotional comforting words (such as “approve,” “adjust,” “ideal,” “desire,” “express,” “try,” “feeling,” “hug,” “warm”) are significantly increased in answer posts. This indicates that in addition to restating the content of question posts, answer posts also use different linguistic styles and techniques, such as expressing emotions, empathy, and providing advice. By observing the content of answer posts, we find that they typically follow four stages: restating the question - expressing understanding - sharing personal experiences - and providing advice. This indicates that social supporters do not express their opinions randomly during the process of providing psychological assistance, but rather respond according to support strategies taught during the counseling training. Therefore, the non-random nature of their answers contributes to a certain level of structural coherence in the content.

The analysis of high-frequency words and topics indicates the presence of linguistic differences among different user posts. Motivated by these findings, we conduct further analysis to explore the linguistic features and differences among posts that received different levels of engagement.

### 3.3 The linguistic features in questions and answers with different engagement

Research in psycholinguistics and computational psychology has demonstrated that differences in the use of vocabulary categories and linguistic style matching can influence the outcomes of counseling or conversation during the process of conversation [44]. Inspired by this, we analyze the linguistic features and differences in the vocabulary categories, linguistic style matching, and language modeling of questions and answers with different engagement.

#### 3.3.1 Definition of high and low engagement

Due to the dominant position of online platforms and the increasing willingness of users to seek online social support, the concept of engagement has become increasingly important. Baldus et al. [45] defined engagement as the behavior of interacting and collaborating with community members. Wu et al. [36] viewed engagement as a mutually dependent process that involves actively contributing personal knowledge, experiences, and cognition to online communities, emphasizing the interactive and bidirectional nature of engagement.

**Figure 1:**
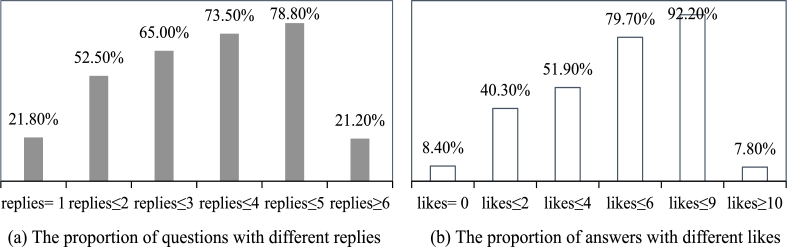
The proportion of questions and answers with different replies and likes.

On the “Yi Xinli” platform, question posts receive varying numbers of replies, all provided by qualified professionals with psychological counseling credentials. Additionally, users have the option to express their recognition of these replies through the “like” label. User replies and “likes” can be considered as interactive and engaging behaviors among users. Building upon existing literature and considering the actual situation on the “Yi Xinli” platform, we utilize the number of replies to question posts and the count of “likes” received by answer posts as indicators to evaluate the level of engagement in questions and answers. We calculate the proportion of question posts with different numbers of replies and answer posts with different numbers of “likes” as shown in Fig. 1.

This study excludes question posts that did not receive any replies, considering them invalid and outside the scope of research. Therefore, the selected question posts must have at least one reply. All replies are provided by professionals with qualifications in psychological counseling, and thus, regardless of whether they receive “like,” they are considered valid answers. To highlight and accentuate the differences in engagement in questions and answers, we have chosen two extreme values with roughly equal quantities. Specifically, questions with replies ≥6 are defined as Questions with High Engagement (shorted as QHE), while questions with replies= 1 are defined as Questions with Low Engagement (QLE). Similarly, answers with likes ≥10 and likes= 0 are selected as Answers with High Engagement (AHE) and Answers with Low Engagement (ALE) respectively.

#### 3.3.2 Vocabulary categories

Previous studies have demonstrated relationships between the dominant usage of certain vocabulary categories with individuals' mental health [27], [28], [29]. Inspired by this, we investigate the different usage of such vocabulary categories in questions and answers with different engagement.

We compare the dominant usage of vocabulary categories in QHE and QLE by using SC-LIWC dictionary [46]. We define the dominance score $SQ_{t}$ of the vocabulary category ***t*** in QHE as the ratio of the proportion of ***t*** in QHE to that in QLE, as shown in formula (1).

(1) $$SQ_{t}=\frac{type{\text{\%}}_{QHE}}{type{\text{\%}}_{QLE}}$$

The closer the value of $SQ_{t}$ is to 1, the more consistent the use of the vocabulary categories between QHE and QLE is. Conversely, the difference is larger in the use of vocabulary categories between QHE and QLE.

Similarly, to explore the differences in vocabulary categories in answer posts with different engagement, we compare the use of SC-LIWC vocabulary categories in AHE and ALE. We define the dominance score $SA_{t}$ of the vocabulary category ***t*** in AHE as the ratio of the proportion of ***t*** in AHE to that in ALE, as shown in formula (2).

(2) $$SA_{t}=\frac{type{\text{\%}}_{AHE}}{type{\text{\%}}_{ALE}}$$

**Figure 2:**
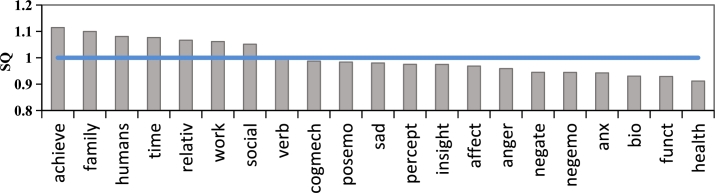
The dominance scores of vocabulary categories in high-engagement question posts.

**Figure 3:**
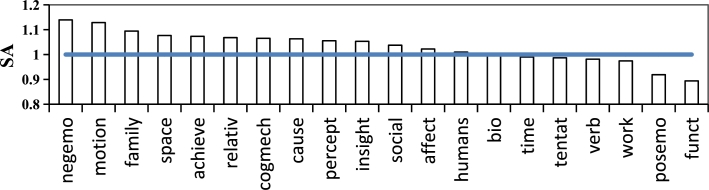
The dominance scores of vocabulary categories in high-engagement answer posts.

The dominance scores of $SQ_{t}$ and $SA_{t}$ on different vocabulary categories are shown in Fig. 2 and Fig. 3. According to Fig. 2, achieve, family, humans, and time words are dominant in QHEs, whereas health, function, biology, and anxiety words are dominant in QLEs. This result indicates that high-engagement question posts are more likely to describe context and reasons, while issues related to emotions and health receive fewer responses. One possible explanation is that focusing solely on emotions may not capture the attention of supporters, and supporters cannot gain a deeper understanding of the seekers, which could limit their ability to offer effective psychological support.

As shown in Fig. 3, AHEs are dominant in negemo(negative emotion), motion, family, and space words. This indicates that answer posts containing these categories of words may receive more likes and higher recognition from users. While ALEs using function and posemo(positive emotion) words have the opposite results. Fig. 3 shows an interesting phenomenon that is contrary to our expectations: the proportion of negative emotion words in high-engagement answer posts is higher, while positive emotional words are lower. There may be two reasons for this phenomenon: firstly, high-engagement answer posts may provide more empathy and restating skills, so the proportion of negative emotion words is higher, which is also reflected in the inclusion of more family, cogmech(cognitive), and cause words; secondly, providing only positive emotions in the form of inspirational quotes may not resonate with help-seekers.

#### 3.3.3 Linguistic style matching

Linguistic Style Matching (LSM) is used to measure the degree of speech matching between conversation partners in psychology [47], [48]. In social networks, the LSM between help-seekers and social supporters can impact the engagement of questions and answers. Conversations with high LSM will result in a stronger willingness to communicate further, while conversations with low LSM may decrease the seeker's willingness to disclose further information and inhibit supportive behaviors from social supporters [49]. To examine the degree of LSM between social supporters and help-seekers, we calculate the LSM between questions and answers under different topics.

LSM calculates scores by counting the functional words used by both parties in the conversation [50]. In English conversations, functional words consist of 9 categories: auxiliary verbs, articles, common adverbs, personal/non-personal pronouns, prepositions, negations, conjunctions, and quantifiers. As Chinese does not have articles, we select the remaining 8 categories. After calculating the percentage of each category ***t*** in the question post ***q*** and the corresponding answer post ***a***, the LSM score on category ***t*** is calculated as Formula (3).

(3) $$LSM_{t}=1-\frac{abs(type{\text{\%}}_{q}-type{\text{\%}}_{a})}{type{\text{\%}}_{q}+type{\text{\%}}_{a}+.0001}$$

Here, ***q*** represents the question post, and ***a*** represents the corresponding answer post. The average LSM score of the 8 categories' functional words is taken as the final LSM score between question ***q*** and answer ***a***.

Based on the topic analysis results in Section 3.2.2, we divide question posts into the corresponding topic set and get the sets of question posts corresponding to the 8 topics. For question posts in each topic, we select the corresponding answer posts with the number of likes ≥10 (AHE) and the number of likes= 0(ALE), and calculate the LSM values between the question post and the corresponding AHE and ALE. The number of question posts, ALE, and AHE in the 8 topics are shown in Table 5. Under all topics, the number of ALE and AHE is relatively balanced.

**Table 5:** The number of posts in 8 topics.

Topic	number of question posts	number of ALE	number of AHE
growth	3040	910	705
marriage	1041	402	270
family	1828	560	540
in-love	3404	1270	861
emotions	1791	516	382
human-relations	2441	747	554
behavioral-therapy	3572	1050	827
career	661	189	130

**Figure 4:**
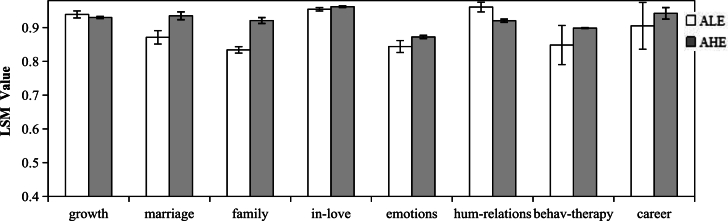
LSM values calculated with non-parametric bootstrap resampling (95% confidence intervals).

Fig. 4 displays the results of the LSM analysis. In most topics, AHEs have higher LSM values with question posts, while ALEs have lower LSM values with question posts. This suggests that answer posts with a higher degree of LSM with question posts are more likely to be recognized by help-seekers and generate positive interactive behaviors and support effects.

The size of the confidence interval indicates that the LSM values of AHE have a smaller confidence interval and are relatively stable, while the LSM values of ALE have a larger confidence interval and fluctuate more. This suggests that high-engagement answer posts have higher consistency with the LSM of the corresponding question posts, whereas the low-engagement answer posts have a larger difference with the LSM of the corresponding question posts, especially in the “career” and “behavioral-therapy” topics. Possible reasons for this are that the content of “career” and “behavioral-therapy” topics has a strong professionalism and specificity, which increases the difficulty of providing support. Most supporters may not be able to cope well with these issues or may have significantly different approaches to them.

Fig. 4 also indicates that in the “growth” and “in-love” topics, the LSM values of AHE and ALE are similar, while in the “human-relations” topic, the LSM value of AHE is lower than that of ALE. The reason for this special result needs further investigation. One possible explanation is that interpersonal problems are complex and cover a wide range of issues related to work, growth, and other areas, which leads to a wide variety of vocabulary categories used in questions and answers, resulting in lower linguistic matching. Similar discussions have been found in existing literature [11], which suggests that interpersonal social problems cover a wide range of social issues, which are less focused and specific compared to other topic issues, resulting in lower linguistic matching.

#### 3.3.4 Language modeling

To gain a deeper comprehension of the differences in language usage about questions and answers with different engagement, we construct a deep learning language model to analyze the differences. The basic idea is to train a language model on one set of data and analyze its perplexity when predicting the other set of data using the trained language model. This enables us to grasp the differences in language modeling between the two sets. Specifically, we use the language model trained on set A to predict set B, and calculate the entropy difference by subtracting the entropy of the model's predictions on set A from the entropy of its predictions on set B. This entropy difference represents the degree of difference in language modeling between sets A and B. The larger the entropy difference, the greater the difference in language usage between the two sets, as shown in Formula (4).

(4) $$E_{A,B}=-(\frac{1}{\left|B_{test}\right|}\sum_{x\in B_{test}}\log(P_{A_{train}}(x))-\frac{1}{\left|A_{test}\right|}\sum_{x\in A_{test}}\log({P_{A}}_{{}_{train}}(x)))$$

$B_{test}$ and $A_{test}$ are the test data for sets B and A respectively. $A_{train}$ is the train data for set A when training the language model.

The Long Short-Term Memory (LSTM) model [51] is a deep learning model based on the RNN that optimizes the problem of short-term memory by adding gate mechanisms. It has been widely used in various tasks such as text classification, text matching, and text prediction. For our study, we select the optimized LSTM model proposed by Merity [52] as the language model. This model incorporates various regularization techniques and achieves high performance and running speed. Additionally, it has low hardware requirements.^4^ The model has been applied in various research studies [53], [54].

To understand the language differences between QLE and QHE, we treat QHE and QLE as A and B in Formula (4), and use QHE to train the LSTM model. We then subtract the entropy predicted by QHE from the entropy predicted by QLE on the model, and the calculated entropy difference represents the degree of language difference between QLE and QHE. Similarly, we use QLE to train the LSTM model and calculate the entropy difference between the entropy values predicted by QHE and QLE on the model, which represents the degree of language difference between QHE and QLE. We conduct the same experiment to understand the language differences between AHE and ALE. The LSTM models trained on QHE, QLE, AHE, and ALE in the above experiments are denoted as $LM_{QHE}$, $LM_{QLE}$, $LM_{AHE}$, and $LM_{ALE}$.

**Figure 5:**
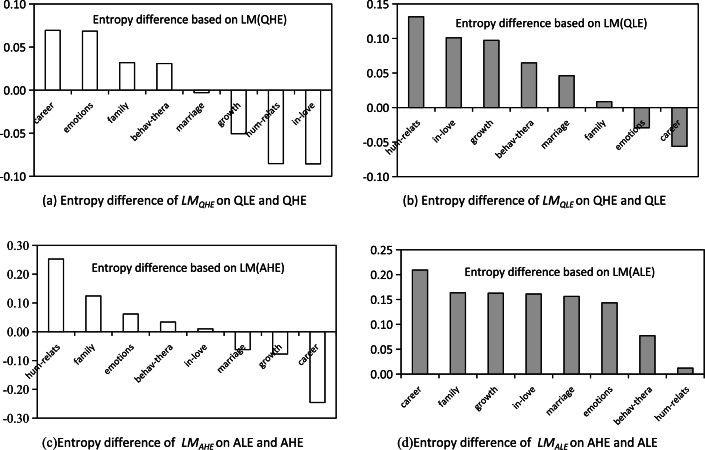
Differences between questions and answers of different engagement in language modeling.

For the experiment, we employ all the words present in each topic as the dictionary and split the data into train, validation, and test sets in an 8:1:1 ratio. The learning rate, dropout, optimizer, and loss function are set to 1e-3, 0.4, SGD, and cross-entropy loss function, respectively. Each language model is trained for 50 epochs. We conducted the same experiment on the 8 topics, and the results are shown in Fig. 5.

Comparing Fig. 5(a) and (b), the entropy difference obtained by the $LM_{QHE}$ model is positive in the “career” and “emotions” topics, indicating that the languages used in these topics have stronger regularity, and are more easily modeled in QHE. Conversely, the “human-relations,” “in-love” and “growth” topics have negative entropy differences, suggesting that the languages used in these topics have lower regularity, and are more difficult to model. The results obtained by the $LM_{QLE}$ model are exactly the opposite, indicating that the regularity of discussing “human-relations,” “in-love” and “growth” topics is stronger in QLE, while the regularity of discussing the “career” and “emotion” topics is stronger in QHE. The “career” and “emotions” topics share commonalities and similar content is more likely to resonate with and attract supporters, resulting in a higher response rate. However, for “in-love,” “human-relations” and “growth” topics, individual differences are more significant, and using similar content may not highlight individual demands, resulting in a lower likelihood of resonance and attention.

Fig. 5(c) indicates that topics such as “human-relations” and “family” are more easily modeled in AHE, as they have a high entropy difference from the model $LM_{AHE}$. However, the entropy difference is negative when predicting the “career” and “growth” topics, indicating that these topics are more difficult to model on $LM_{AHE}$. This suggests that there is greater regularity and commonality in the responses related to topics such as “human-relations” and “family” in AHE, while there is greater variability in the responses related to topics such as “career” and “growth”.

According to Fig. 5(d), the entropy difference from the $LM_{ALE}$ model is positive and generally higher in all topics. This suggests that the content of ALE has a higher degree of commonality in all topics. One possible reason for this commonality is that ALE does not provide carefully tailored responses to the seeker's questions, but rather a patchwork of answers, resulting in high similarity between the content of answer posts that cannot be recognized by users. Comparing Fig. 5(c) and (d), it can be observed that the largest entropy difference value is for the “career” topic, indicating that the phenomenon of similar content in ALE is more severe when replying to the “career” topic.

## 4 Discussion and future work

Previous research has primarily focused on exploring the behaviors and language of users in social media related to mental health, such as the influence of help-seeker behaviors and language on obtaining social support [6], [26], as well as the support effects and influencing factors of different categories of social supporters, including regular users and professional counselors [11], [55]. However, there is little research on the linguistic features and interactive behaviors of users on specialized online platforms for professional mental health services. In these platforms, help-seekers typically disclose issues related to emotional distress, while social supporters are qualified mental health professionals. Therefore, there may be differences in language expression and interaction among users. In this study, we use text mining and deep learning methods to analyze the question-and-answer posts from the Chinese online mental health community “Yi Xinli”. We specifically focus on investigating the linguistic features and differences in the posts with varying levels of engagement. The objective is to understand the linguistic features present in posts that promote user engagement and interaction on the professional platform, so as to gain deeper insights into user behavior and factors influencing counseling effectiveness on the platform. Our analysis yielded several valuable findings.

We found that the majority of help seekers were concerned and troubled by work-related issues, and we roughly classified the question topics into eight categories. In contrast to previous literature [11], [33], our findings suggest that the problems presented by users on the platform are not predominantly related to mental disorders such as depression, anxiety, or post-traumatic stress disorder. Instead, they often express everyday life stressors and emotional difficulties, highlighting the diversity of user needs on the platform. Furthermore, we observed that social supporters employed specific support techniques, such as empathy, advice, and support, in their responses. These findings align with Hill's counseling conversation theory [56], suggesting that social supporters follow the support strategies taught in counseling training rather than expressing content randomly.

Jiang et al. [29] found in their study on the Chinese health community “Tianmi Jiayuan” that the use of health-related vocabulary was negatively correlated with the receipt of informational social support by help seekers. Our study reached a similar conclusion: high-engagement question posts often exhibited higher frequencies of achievement, family, human, and time-related words, while the use of health, biology, and anxiety-related words was relatively low. We attribute this finding to the cautious nature of social supporters on Chinese online mental health platforms, as they refrain from providing excessive responses and attention to issues involving diseases and emotions without a comprehensive understanding of individual personalities and the severity of the condition. Additionally, we made an unexpected but interesting discovery: high-engagement answer posts displayed a higher proportion of negative emotion words and a lower proportion of positive emotion words. We attribute this result to the increased use of empathy and paraphrasing support techniques in high-engagement answer posts, leading to a higher proportion of negative emotion words. This differs from the findings of previous studies conducted on English-speaking mental health-related communities [30], [57], which indicated that high-engagement answers often employed supportive language associated with encouragement, support, and reflective listening. Thus, it can be inferred that social supporters from different cultural backgrounds exhibit differences in the use of support strategies and techniques. This finding can provide insights for research on social support in diverse cultural contexts.

Previous research has employed LSM to comprehend the interactive effects between social supporters and help-seekers [49], [50]. Perez et al. [49] discovered that counseling sessions with high engagement exhibited higher LSM between supporters and help-seekers, while Nobles et al. [50] yielded contrasting results. Perez et al. attributed this disparity to the different counseling modalities employed, with their study focusing on face-to-face interactions, whereas Nobles et al.'s research was based on message exchanges. Our study represents the first application of LSM calculations in the context of Chinese online mental health counseling. The research findings indicate that, in the majority of topics, there is a higher LSM between high-engagement answers and questions. This not only validates the greater linguistic consistency between high-engagement answers and questions on Chinese online mental health platforms but also suggests that the interaction outcomes between help-seekers and social supporters resemble face-to-face counseling sessions. This finding partially substantiates the effectiveness of online mental health support as a novel mode of assistance, enabling platform administrators to enhance the quality of social supporters to achieve better support outcomes and provide improved services.

By employing an LSTM model to analyze and model questions and answers of different engagement, we discovered that high-engagement question posts exhibited greater regularity and commonality in the topics of “career” and “emotions.” This could be attributed to the fact that these topics involve more universal and common issues, leading users to employ similar language expressions. On the other hand, greater variability and irregularity were observed in questions related to “in-love,” “human-relations,” and “growth.” This may be due to the diverse and individualized nature of these topics, resulting in greater disparities and weaker regularity. Similarly, high-engagement answer posts displayed more regularity and commonality in responses related to “human-relations” and “family” topics. This could be because these topics encompass issues and relationships that are commonly faced by people, thus generating more standardized response patterns. Conversely, responses related to “career” and “growth” exhibited greater disparities and weaker regularity, as these topics involve personal experiences and development, leading to varying perspectives and experiences among different respondents. Additionally, we observed a significant occurrence of content similarity among low-engagement answer posts across all topics. This indicates a high likelihood of replication and plagiarism in low-engagement answers. This could be attributed to some supporters lacking professional knowledge or creativity, resulting in a tendency to simply replicate other answers or provide template-like responses.

Through our preliminary exploration, we have gained initial insights into the focus points and topic scope of the question-and-answer posts, the characteristics, and disparities in content among posts with different engagement on Chinese online mental health counseling platforms. We find that users of online mental health counseling platforms are not passive participants. Instead, the process of help-seekers posting questions and social supporters providing answers is a dynamic one that involves reflection and autonomy. Social supporters employ different discourse strategies to offer higher quality and more targeted information and resources, while help-seekers can gain more attention and assistance through various linguistic styles. Additionally, there are differences in support strategies used by social supporters from different cultural backgrounds. The findings of this study validate the effectiveness of online mental health platforms as tools for psychological support, revealing users' needs and behavioral patterns on the platform, and providing valuable references for further research on question-answer interactions on Chinese online mental health counseling platforms. Moreover, these findings have positive implications for advancing the practice of online mental health counseling. They also hold significant guidance for improving online mental health counseling services, enhancing the support skills of social supporters, and better meeting the needs of help-seekers.

Future research will delve deeper from multiple perspectives to investigate the causes of health issues reflected in question posts and the severity of psychological crises, providing a more comprehensive understanding of help-seeker situations. Additionally, we will analyze factors that influence answer engagement from various aspects, including social supporters' professional backgrounds, experiences, and linguistic characteristics, aiming to analyze the influencing factors of post-engagement from a more scientific and comprehensive perspective. Furthermore, we plan to establish a deep learning model for support strategies to examine the regularity of strategy usage by social supporters, the relationship between strategies and questions' topics, the impact of strategies on help-seekers emotional changes, and the interactive effects between help-seekers and social supporters. This will enable us to gain a deeper understanding of the interaction patterns and influencing factors between help-seekers and social supporters in online mental health communities.

## 5 Limitations and ethical considerations

This study has some limitations. Firstly, the analysis heavily relies on the SC-LIWC vocabulary. Although it helps discover the differences and categories of vocabulary in the questions and answers, it cannot explain the reasons behind these differences. Secondly, there may be additional topics in question posts that require further detailed and in-depth research beyond the ones summarized in this paper. Finally, since the data set used in the study is from the “Yi Xin Li” online mental health counseling platform, the conclusions may be heavily platform-dependent. Therefore, users must carefully examine the content and quality of counseling in actual applications.

This work does not offer any recommendations related to treatment or diagnosis. The dataset is from publicly available and anonymous posts in the “Yi Xin Li” platform and is strictly limited to scientific research use. Personal information, such as nicknames, personal information, phone numbers, and URL links, was removed during the data construction process. Due to copyright issues, the dataset is not currently open source, but we will negotiate with the “Yi Xin Li” platform to make it open source within the scope of legal permission.

## 6 Conclusion

To understand the content and features of question-and-answer posts in the Chinese online mental health platform, this study utilized text mining and deep learning techniques to analyze high-frequency words and topics, capturing the focal points of the posts and the range of topics covered. Building upon this analysis, the study further examined the features and differences of question-and-answer posts with varying engagement from three perspectives: vocabulary categories, linguistic style matching, and language modeling, and found important features and patterns exhibited by high-engagement questions and answers. This research contributes to understanding the language use and behavioral features of help-seekers and social supporters on Chinese online mental health platforms, providing valuable information for a deeper understanding of user needs and the quality of support provided by social supporters. In future research, we will incorporate more deep learning models and explore the interaction content and effects between help-seekers and social supporters from multiple aspects, including seekers' psychological health reasons, backgrounds, crisis levels, as well as supporters' experiences and professional expertise. This will offer a more comprehensive perspective to analyze the interaction patterns and dynamics among users in online mental health platforms.

## CRediT authorship contribution statement

**Liyuan Zhang:** Conceptualization, Data curation, Formal analysis, Investigation, Methodology, Resources, Writing – original draft. **Dexi Liu:** Funding acquisition, Project administration, Software, Supervision, Validation. **Jing Li:** Validation, Writing – review & editing. **Changxuan Wan:** Visualization, Writing – review & editing. **Xiping Liu:** Validation, Writing – review & editing.

## Declaration of Competing Interest

The authors declare that they have no known competing financial interests or personal relationships that could have appeared to influence the work reported in this paper.

## Figures and Tables

**Figure:**
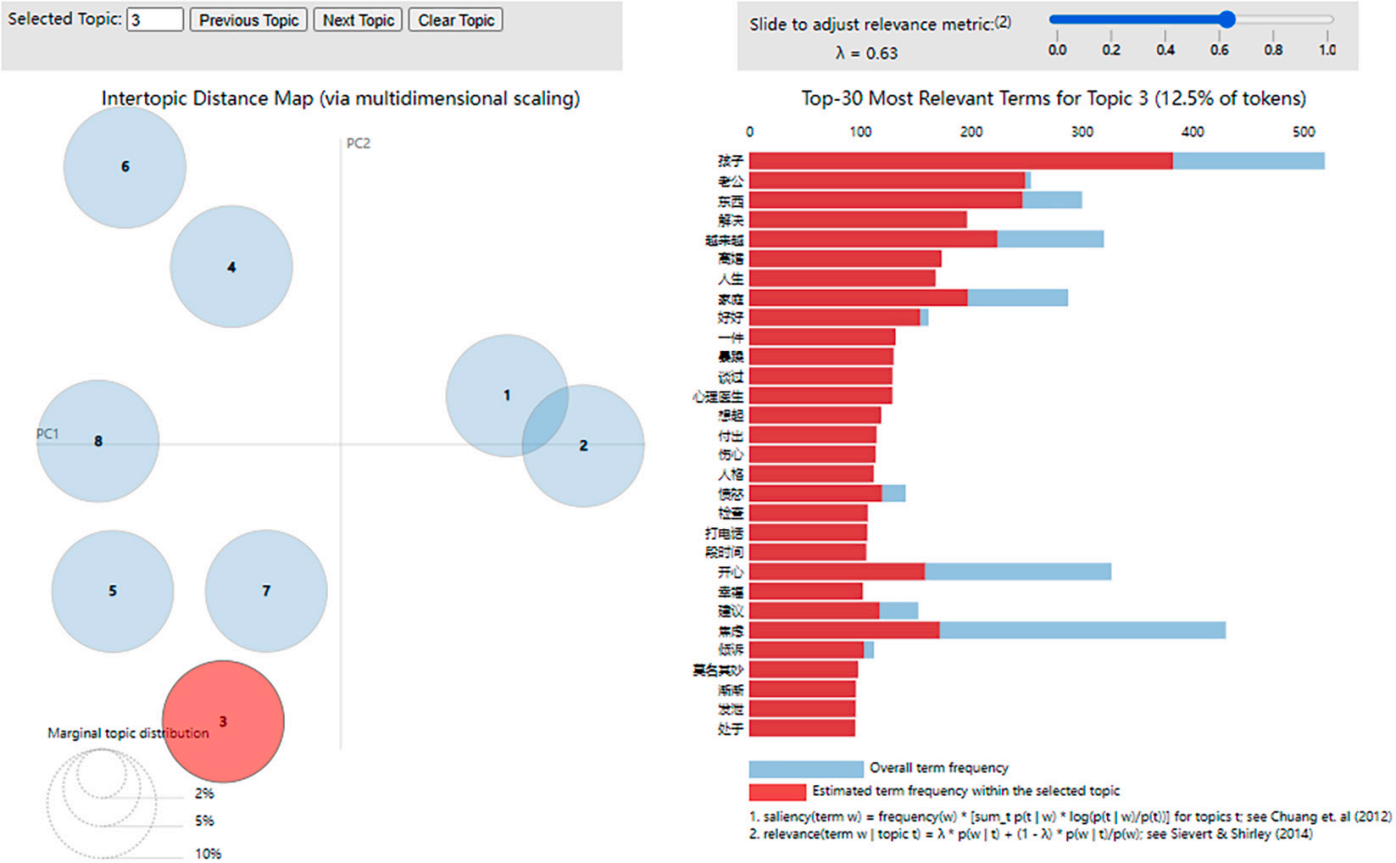


**Figure:**
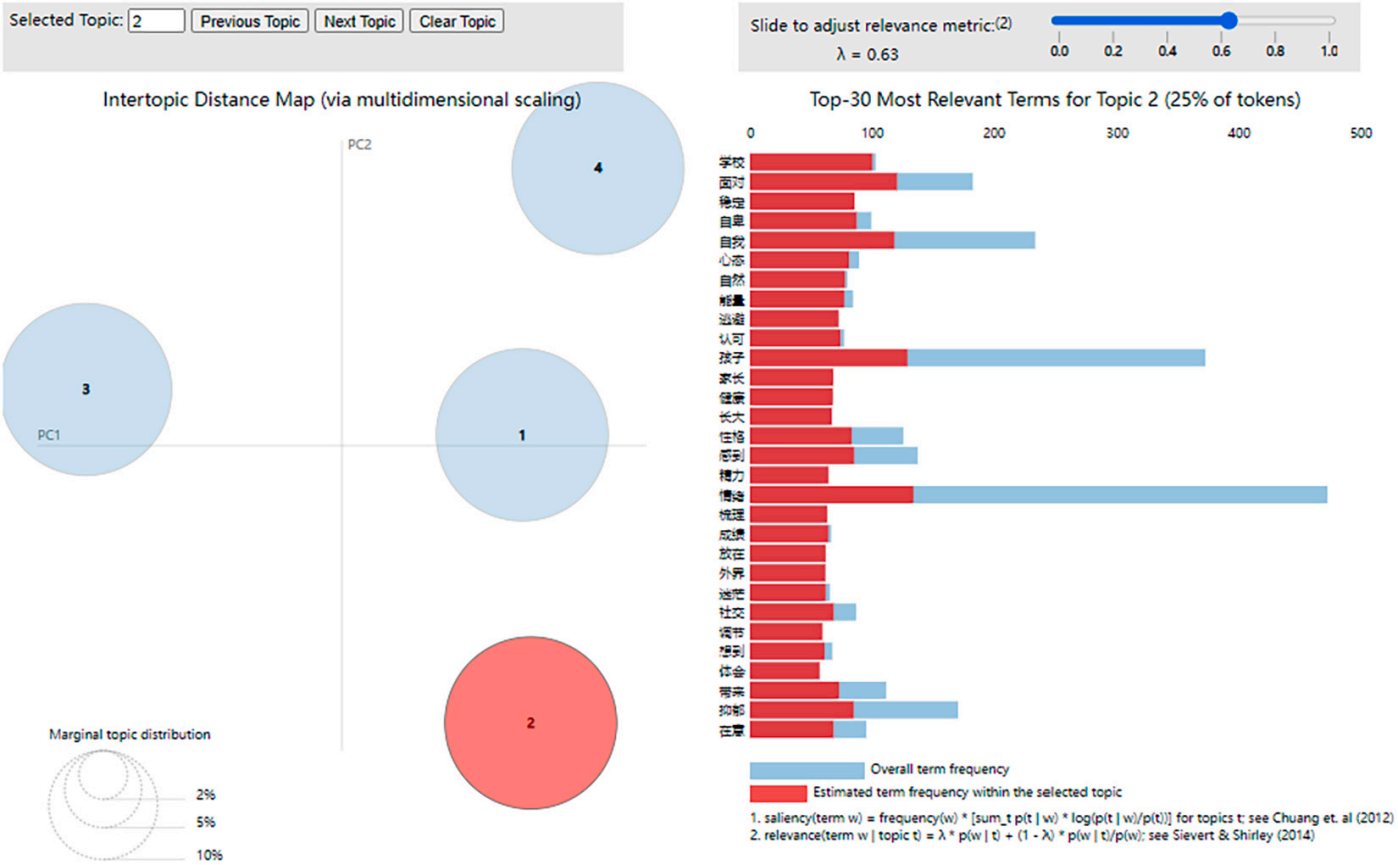


**Figure:**
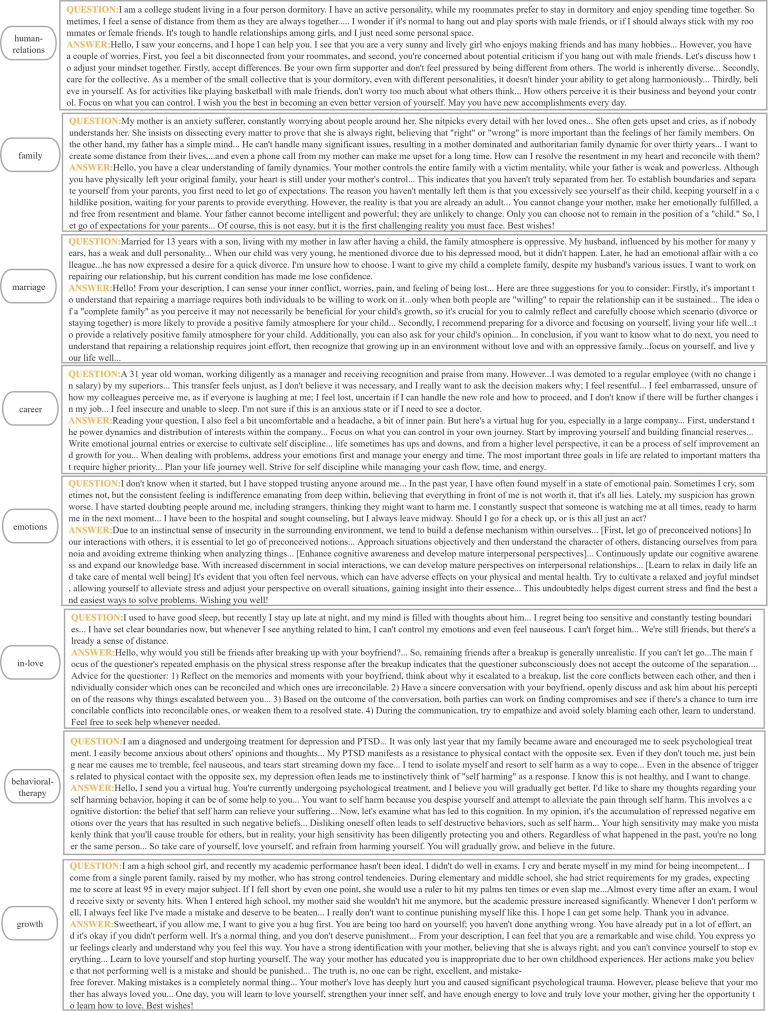


## Data Availability

The data sets generated and analyzed during this study are available from the corresponding author upon reasonable request.

## Footnotes

1 https://www.xinli001.com/qa?source=pc-home.

2 http://www.cn-psy.com/.

3 https://github.com/fxsjy/jieba.

4 https://github.com/salesforce/awd-lstm-lm.
